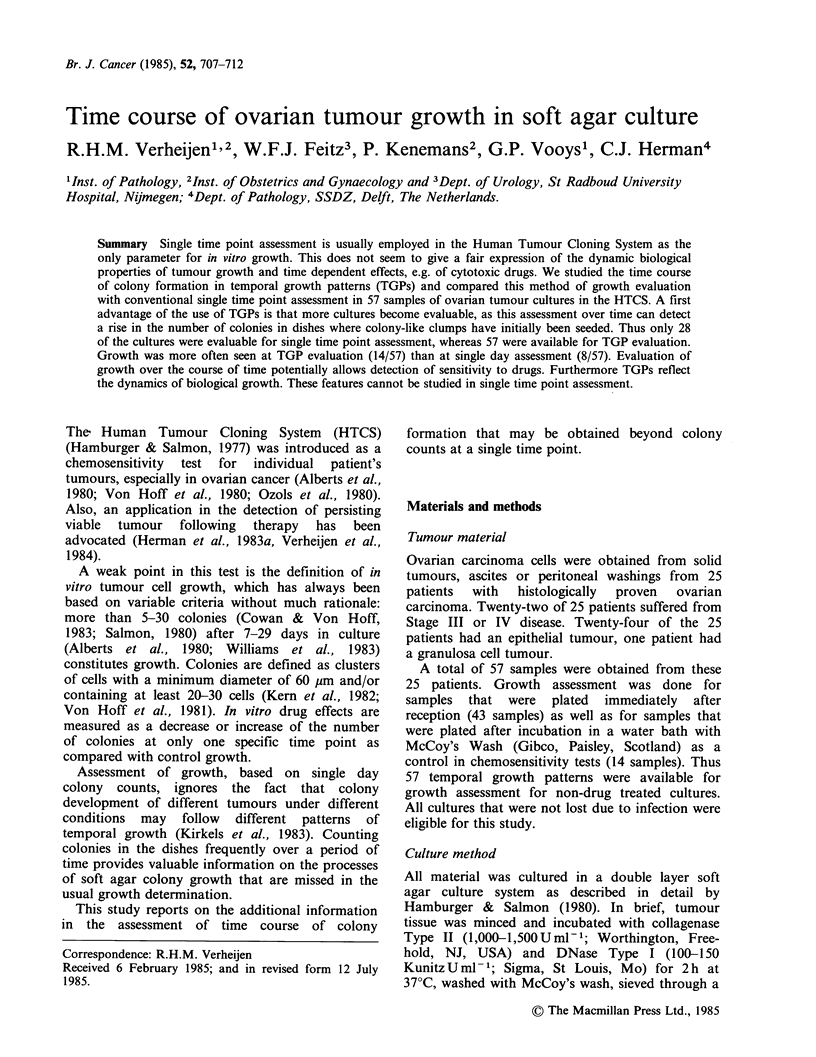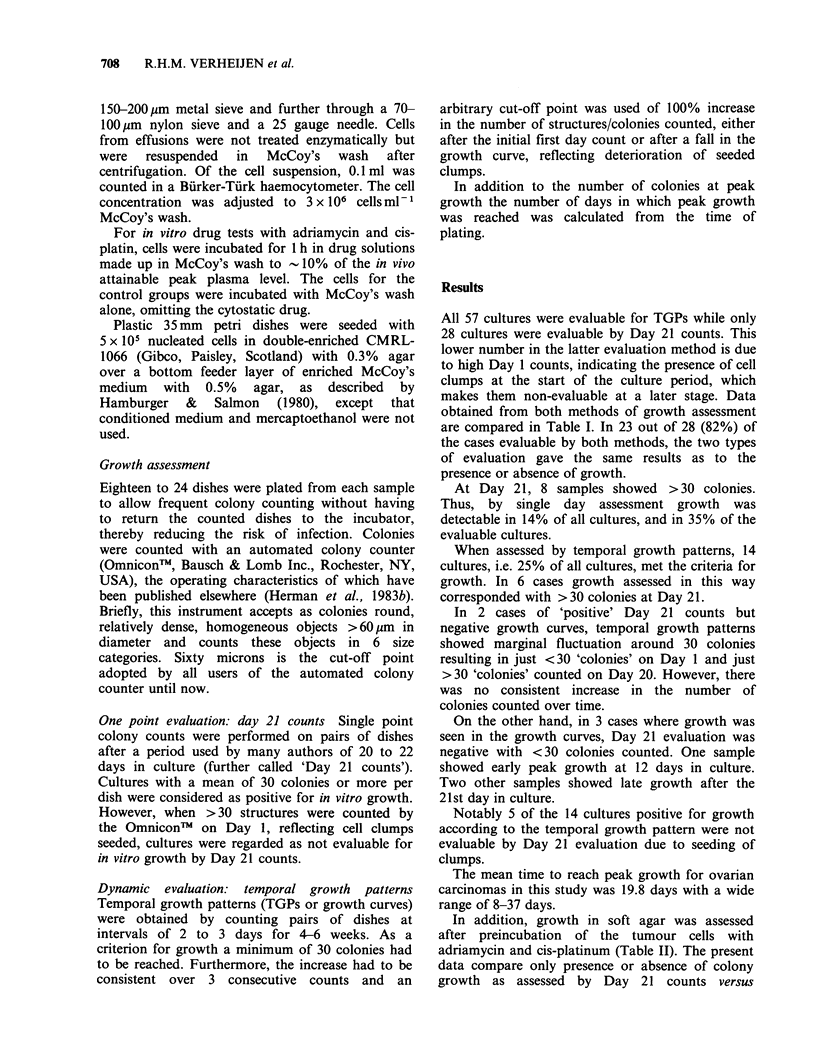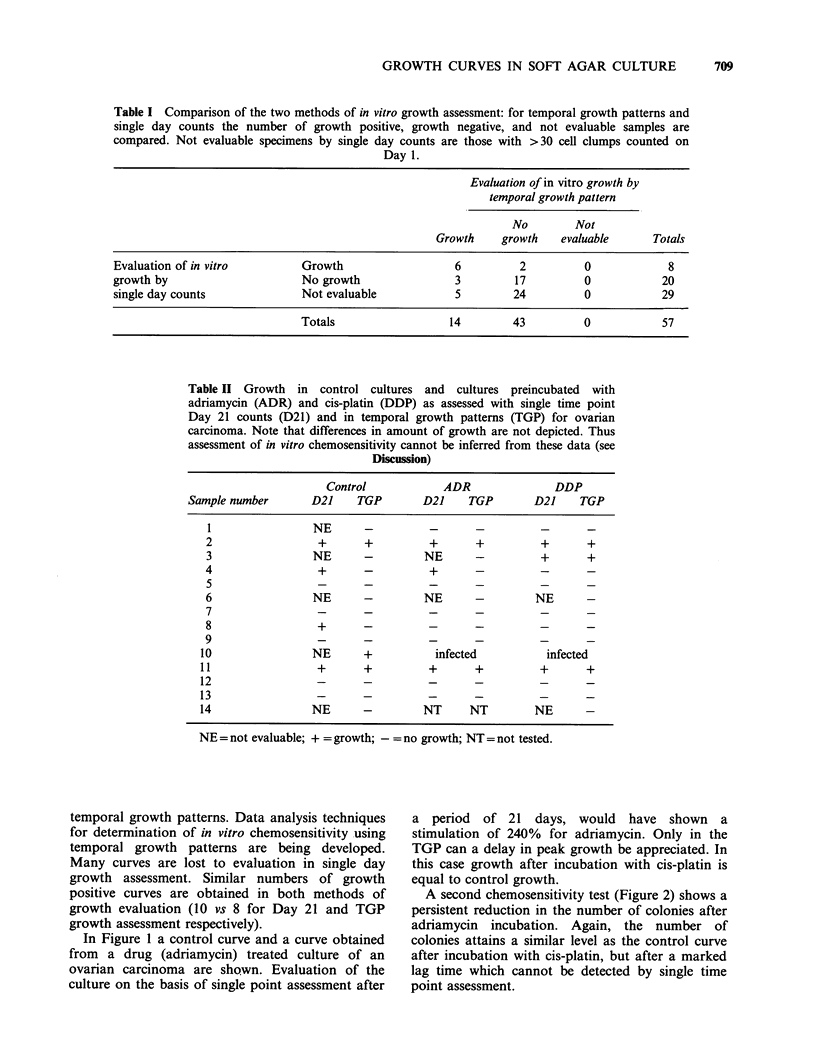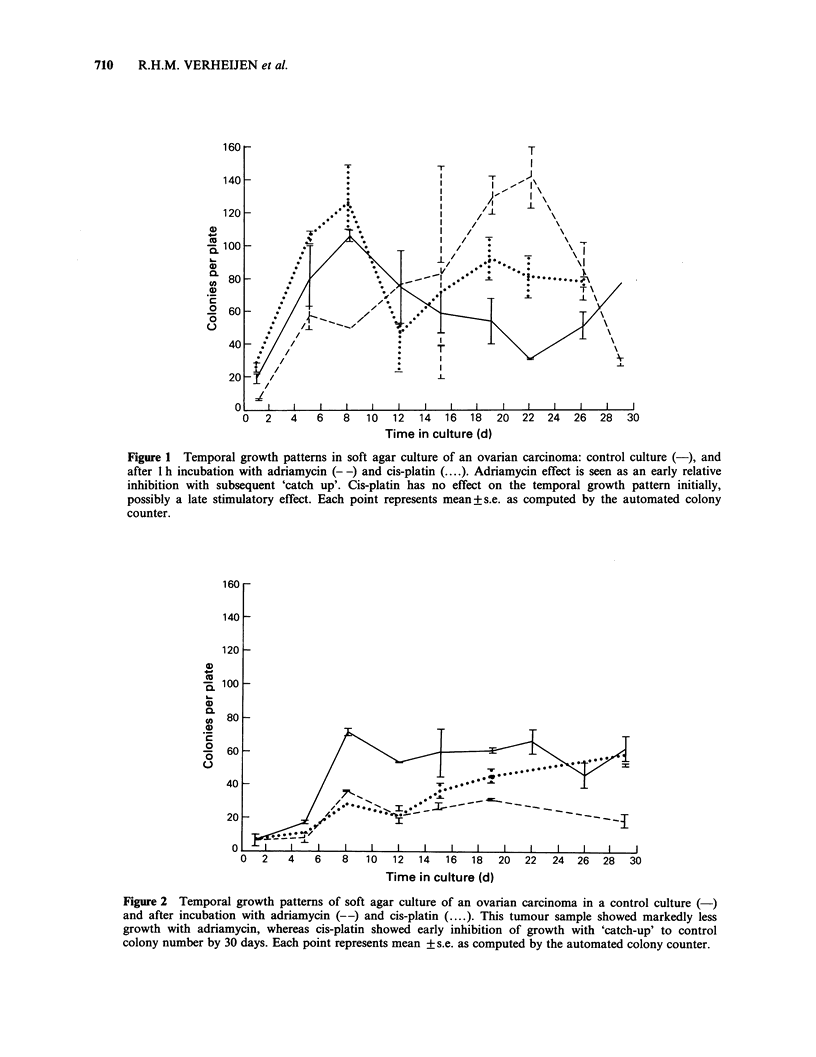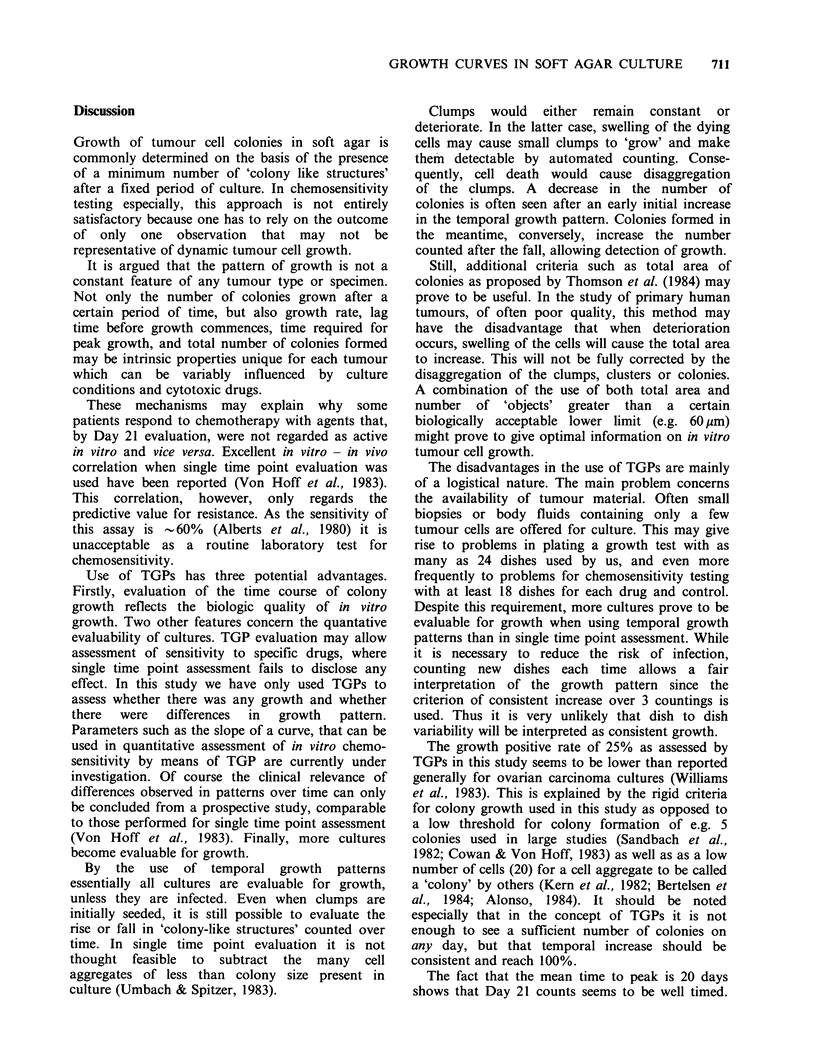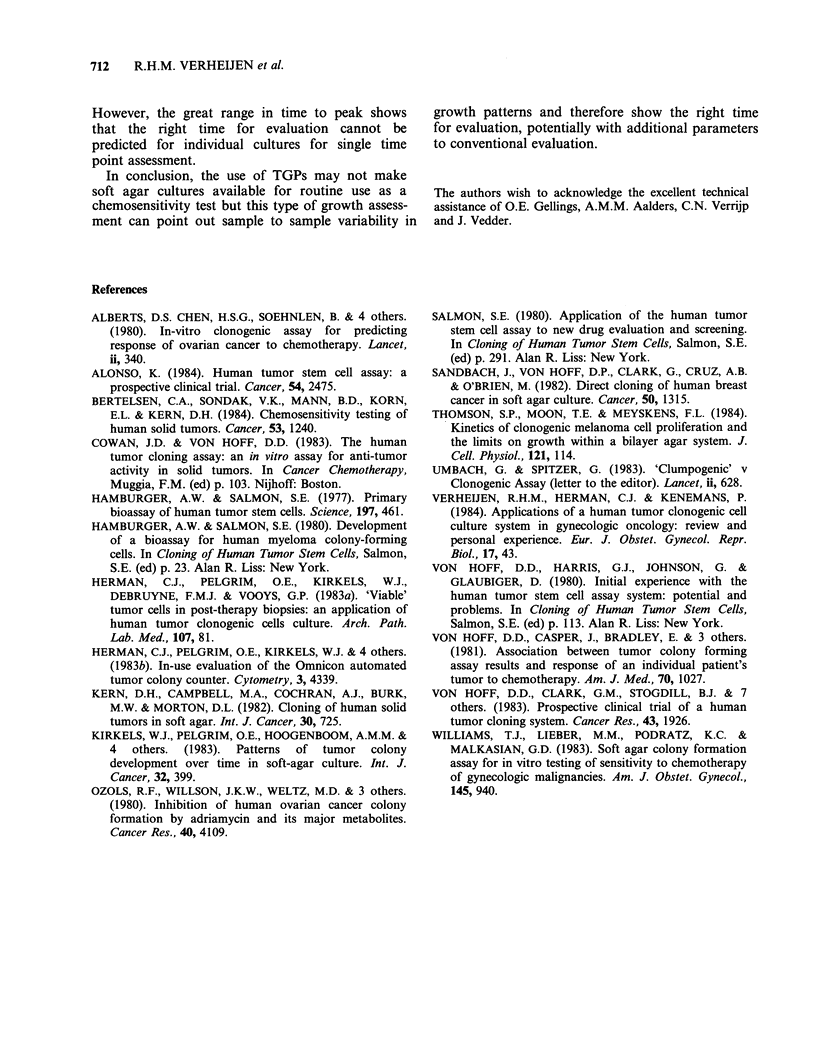# Time course of ovarian tumour growth in soft agar culture.

**DOI:** 10.1038/bjc.1985.247

**Published:** 1985-11

**Authors:** R. H. Verheijen, W. F. Feitz, P. Kenemans, G. P. Vooys, C. J. Herman

## Abstract

Single time point assessment is usually employed in the Human Tumour Cloning System as the only parameter for in vitro growth. This does not seem to give a fair expression of the dynamic biological properties of tumour growth and time dependent effects, e.g. of cytotoxic drugs. We studied the time course of colony formation in temporal growth patterns (TGPs) and compared this method of growth evaluation with conventional single time point assessment in 57 samples of ovarian tumour cultures in the HTCS. A first advantage of the use of TGPs is that more cultures become evaluable, as this assessment over time can detect a rise in the number of colonies in dishes where colony-like clumps have initially been seeded. Thus only 28 of the cultures were evaluable for single time point assessment, whereas 57 were available for TGP evaluation. Growth was more often seen at TGP evaluation (14/57) than at single day assessment (8/57). Evaluation of growth over the course of time potentially allows detection of sensitivity to drugs. Furthermore TGPs reflect the dynamics of biological growth. These features cannot be studied in single time point assessment.


*Br. J. Cancer* (1985), **52**, 707–712

### Time course of ovarian tumour growth in soft agar culture

R.H.M. Verheijen[1,2], W.F.J. Feitz[3], P. Kenemans[2], G.P. Vooys[1], C.J. Herman[4]

[1]*Inst. of Pathology,* [2]*Inst. of Obstetrics and Gynaecology and* [3]*Dept. of Urology, St Radboud University Hospital, Nijmegen;* [4]*Dept. of Pathology, SSDZ, Delft, The Netherlands.*

**Summary** Single time point assessment is usually employed in the Human Tumour Cloning System as the only parameter for *in vitro* growth. This does not seem to give a fair expression of the dynamic biological properties of tumour growth and time dependent effects, e.g. of cytotoxic drugs. We studied the time course of colony formation in temporal growth patterns (TGPs) and compared this method of growth evaluation with conventional single time point assessment in 57 samples of ovarian tumour cultures in the HTCS. A first advantage of the use of TGPs is that more cultures become evaluable, as this assessment over time can detect a rise in the number of colonies in dishes where colony-like clumps have initially been seeded. Thus only 28 of the cultures were evaluable for single time point assessment, whereas 57 were available for TGP evaluation. Growth was more often seen at TGP evaluation (14/57) than at single day assessment (8/57). Evaluation of growth over the course of time potentially allows detection of sensitivity to drugs. Furthermore TGPs reflect the dynamics of biological growth. These features cannot be studied in single time point assessment.

The Human Tumour Cloning System (HTCS) (Hamburger & Salmon, 1977) was introduced as a chemosensitivity test for individual patient's tumours, especially in ovarian cancer (Alberts *et al.*, 1980; Von Hoff *et al.*, 1980; Ozols *et al.*, 1980). Also, an application in the detection of persisting viable tumour following therapy has been advocated (Herman *et al.*, 1983*a*, Verheijen *et al.*, 1984).

A weak point in this test is the definition of *in vitro* tumour cell growth, which has always been based on variable criteria without much rationale: more than 5–30 colonies (Cowan & Von Hoff, 1983; Salmon, 1980) after 7–29 days in culture (Alberts *et al.*, 1980; Williams *et al.*, 1983) constitutes growth. Colonies are defined as clusters of cells with a minimum diameter of 60 $\mu$m and/or containing at least 20–30 cells (Kern *et al.*, 1982; Von Hoff *et al.*, 1981). *In vitro* drug effects are measured as a decrease or increase of the number of colonies at only one specific time point as compared with control growth.

Assessment of growth, based on single day colony counts, ignores the fact that colony development of different tumours under different conditions may follow different patterns of temporal growth (Kirkels *et al.*, 1983). Counting colonies in the dishes frequently over a period of time provides valuable information on the processes of soft agar colony growth that are missed in the usual growth determination.

This study reports on the additional information in the assessment of time course of colony formation that may be obtained beyond colony counts at a single time point.

Correspondence: R.H.M. Verheijen
Received 6 February 1985; and in revised form 12 July 1985.

### Materials and methods

### *Tumour material*

Ovarian carcinoma cells were obtained from solid tumours, ascites or peritoneal washings from 25 patients with histologically proven ovarian carcinoma. Twenty-two of 25 patients suffered from Stage III or IV disease. Twenty-four of the 25 patients had an epithelial tumour, one patient had a granulosa cell tumour.

A total of 57 samples were obtained from these 25 patients. Growth assessment was done for samples that were plated immediately after reception (43 samples) as well as for samples that were plated after incubation in a water bath with McCoy's Wash (Gibco, Paisley, Scotland) as a control in chemosensitivity tests (14 samples). Thus 57 temporal growth patterns were available for growth assessment for non-drug treated cultures. All cultures that were not lost due to infection were eligible for this study.

### *Culture method*

All material was cultured in a double layer soft agar culture system as described in detail by Hamburger & Salmon (1980). In brief, tumour tissue was minced and incubated with collagenase Type II (1,000–1,500 U $ml^{-1}$; Worthington, Freehold, NJ, USA) and DNase Type I (100–150 Kunitz U $ml^{-1}$; Sigma, St Louis, Mo) for 2 h at 37°C, washed with McCoy's wash, sieved through a

© The Macmillan Press Ltd., 1985

150–200 μm metal sieve and further through a 70–100 μm nylon sieve and a 25 gauge needle. Cells from effusions were not treated enzymatically but were resuspended in McCoy's wash after centrifugation. Of the cell suspension, 0.1 ml was counted in a Bürker-Türk haemocytometer. The cell concentration was adjusted to $3 \times 10^6$ cells $ml^{-1}$ McCoy's wash.

For *in vitro* drug tests with adriamycin and cis-platin, cells were incubated for 1 h in drug solutions made up in McCoy's wash to ~10% of the *in vivo* attainable peak plasma level. The cells for the control groups were incubated with McCoy's wash alone, omitting the cytostatic drug.

Plastic 35 mm petri dishes were seeded with $5 \times 10^5$ nucleated cells in double-enriched CMRL-1066 (Gibco, Paisley, Scotland) with 0.3% agar over a bottom feeder layer of enriched McCoy's medium with 0.5% agar, as described by Hamburger & Salmon (1980), except that conditioned medium and mercaptoethanol were not used.

*Growth assessment*

Eighteen to 24 dishes were plated from each sample to allow frequent colony counting without having to return the counted dishes to the incubator, thereby reducing the risk of infection. Colonies were counted with an automated colony counter (Omnicon™, Bausch & Lomb Inc., Rochester, NY, USA), the operating characteristics of which have been published elsewhere (Herman *et al.*, 1983*b*). Briefly, this instrument accepts as colonies round, relatively dense, homogeneous objects >60 μm in diameter and counts these objects in 6 size categories. Sixty microns is the cut-off point adopted by all users of the automated colony counter until now.

*One point evaluation: day 21 counts* Single point colony counts were performed on pairs of dishes after a period used by many authors of 20 to 22 days in culture (further called 'Day 21 counts'). Cultures with a mean of 30 colonies or more per dish were considered as positive for *in vitro* growth. However, when >30 structures were counted by the Omnicon™ on Day 1, reflecting cell clumps seeded, cultures were regarded as not evaluable for *in vitro* growth by Day 21 counts.

*Dynamic evaluation: temporal growth patterns* Temporal growth patterns (TGPs or growth curves) were obtained by counting pairs of dishes at intervals of 2 to 3 days for 4–6 weeks. As a criterion for growth a minimum of 30 colonies had to be reached. Furthermore, the increase had to be consistent over 3 consecutive counts and an arbitrary cut-off point was used of 100% increase in the number of structures/colonies counted, either after the initial first day count or after a fall in the growth curve, reflecting deterioration of seeded clumps.

In addition to the number of colonies at peak growth the number of days in which peak growth was reached was calculated from the time of plating.

### Results

All 57 cultures were evaluable for TGPs while only 28 cultures were evaluable by Day 21 counts. This lower number in the latter evaluation method is due to high Day 1 counts, indicating the presence of cell clumps at the start of the culture period, which makes them non-evaluable at a later stage. Data obtained from both methods of growth assessment are compared in Table I. In 23 out of 28 (82%) of the cases evaluable by both methods, the two types of evaluation gave the same results as to the presence or absence of growth.

At Day 21, 8 samples showed >30 colonies. Thus, by single day assessment growth was detectable in 14% of all cultures, and in 35% of the evaluable cultures.

When assessed by temporal growth patterns, 14 cultures, i.e. 25% of all cultures, met the criteria for growth. In 6 cases growth assessed in this way corresponded with >30 colonies at Day 21.

In 2 cases of 'positive' Day 21 counts but negative growth curves, temporal growth patterns showed marginal fluctuation around 30 colonies resulting in just <30 'colonies' on Day 1 and just >30 'colonies' counted on Day 20. However, there was no consistent increase in the number of colonies counted over time.

On the other hand, in 3 cases where growth was seen in the growth curves, Day 21 evaluation was negative with <30 colonies counted. One sample showed early peak growth at 12 days in culture. Two other samples showed late growth after the 21st day in culture.

Notably 5 of the 14 cultures positive for growth according to the temporal growth pattern were not evaluable by Day 21 evaluation due to seeding of clumps.

The mean time to reach peak growth for ovarian carcinomas in this study was 19.8 days with a wide range of 8–37 days.

In addition, growth in soft agar was assessed after preincubation of the tumour cells with adriamycin and cis-platinum (Table II). The present data compare only presence or absence of colony growth as assessed by Day 21 counts *versus*

**Table I** Comparison of the two methods of *in vitro* growth assessment: for temporal growth patterns and single day counts the number of growth positive, growth negative, and not evaluable samples are compared. Not evaluable specimens by single day counts are those with >30 cell clumps counted on Day 1.

| | | *Evaluation of* in vitro *growth by temporal growth pattern* | | | |
|---|---|---|---|---|---|
| | | *Growth* | *No growth* | *Not evaluable* | *Totals* |
| Evaluation of *in vitro* growth by single day counts | Growth | 6 | 2 | 0 | 8 |
| | No growth | 3 | 17 | 0 | 20 |
| | Not evaluable | 5 | 24 | 0 | 29 |
| | Totals | 14 | 43 | 0 | 57 |

**Table II** Growth in control cultures and cultures preincubated with adriamycin (ADR) and cis-platin (DDP) as assessed with single time point Day 21 counts (D21) and in temporal growth patterns (TGP) for ovarian carcinoma. Note that differences in amount of growth are not depicted. Thus assessment of *in vitro* chemosensitivity cannot be inferred from these data (see **Discussion**)

| | *Control* | | *ADR* | | *DDP* | |
|---|---|---|---|---|---|---|
| *Sample number* | *D21* | *TGP* | *D21* | *TGP* | *D21* | *TGP* |
| 1 | NE | – | – | – | – | – |
| 2 | + | + | + | + | + | + |
| 3 | NE | – | NE | – | + | + |
| 4 | + | – | + | – | – | – |
| 5 | – | – | – | – | – | – |
| 6 | NE | – | NE | – | NE | – |
| 7 | – | – | – | – | – | – |
| 8 | + | – | – | – | – | – |
| 9 | – | – | – | – | – | – |
| 10 | NE | + | infected | | infected | |
| 11 | + | + | + | + | + | + |
| 12 | – | – | – | – | – | – |
| 13 | – | – | – | – | – | – |
| 14 | NE | – | NT | NT | NE | – |

NE = not evaluable; + = growth; – = no growth; NT = not tested.

temporal growth patterns. Data analysis techniques for determination of *in vitro* chemosensitivity using temporal growth patterns are being developed. Many curves are lost to evaluation in single day growth assessment. Similar numbers of growth positive curves are obtained in both methods of growth evaluation (10 *vs* 8 for Day 21 and TGP growth assessment respectively).

In Figure 1 a control curve and a curve obtained from a drug (adriamycin) treated culture of an ovarian carcinoma are shown. Evaluation of the culture on the basis of single point assessment after a period of 21 days, would have shown a stimulation of 240% for adriamycin. Only in the TGP can a delay in peak growth be appreciated. In this case growth after incubation with cis-platin is equal to control growth.

A second chemosensitivity test (Figure 2) shows a persistent reduction in the number of colonies after adriamycin incubation. Again, the number of colonies attains a similar level as the control curve after incubation with cis-platin, but after a marked lag time which cannot be detected by single time point assessment.

**Figure 1** Temporal growth patterns in soft agar culture of an ovarian carcinoma: control culture (—), and after 1 h incubation with adriamycin (– –) and cis-platin (....). Adriamycin effect is seen as an early relative inhibition with subsequent 'catch up'. Cis-platin has no effect on the temporal growth pattern initially, possibly a late stimulatory effect. Each point represents mean ± s.e. as computed by the automated colony counter.

**Figure 2** Temporal growth patterns of soft agar culture of an ovarian carcinoma in a control culture (—) and after incubation with adriamycin (––) and cis-platin (....). This tumour sample showed markedly less growth with adriamycin, whereas cis-platin showed early inhibition of growth with 'catch-up' to control colony number by 30 days. Each point represents mean ± s.e. as computed by the automated colony counter.

### Discussion

Growth of tumour cell colonies in soft agar is commonly determined on the basis of the presence of a minimum number of 'colony like structures' after a fixed period of culture. In chemosensitivity testing especially, this approach is not entirely satisfactory because one has to rely on the outcome of only one observation that may not be representative of dynamic tumour cell growth.

It is argued that the pattern of growth is not a constant feature of any tumour type or specimen. Not only the number of colonies grown after a certain period of time, but also growth rate, lag time before growth commences, time required for peak growth, and total number of colonies formed may be intrinsic properties unique for each tumour which can be variably influenced by culture conditions and cytotoxic drugs.

These mechanisms may explain why some patients respond to chemotherapy with agents that, by Day 21 evaluation, were not regarded as active *in vitro* and *vice versa*. Excellent *in vitro* – *in vivo* correlation when single time point evaluation was used have been reported (Von Hoff *et al.*, 1983). This correlation, however, only regards the predictive value for resistance. As the sensitivity of this assay is ~60% (Alberts *et al.*, 1980) it is unacceptable as a routine laboratory test for chemosensitivity.

Use of TGPs has three potential advantages. Firstly, evaluation of the time course of colony growth reflects the biologic quality of *in vitro* growth. Two other features concern the quantative evaluability of cultures. TGP evaluation may allow assessment of sensitivity to specific drugs, where single time point assessment fails to disclose any effect. In this study we have only used TGPs to assess whether there was any growth and whether there were differences in growth pattern. Parameters such as the slope of a curve, that can be used in quantitative assessment of *in vitro* chemosensitivity by means of TGP are currently under investigation. Of course the clinical relevance of differences observed in patterns over time can only be concluded from a prospective study, comparable to those performed for single time point assessment (Von Hoff *et al.*, 1983). Finally, more cultures become evaluable for growth.

By the use of temporal growth patterns essentially all cultures are evaluable for growth, unless they are infected. Even when clumps are initially seeded, it is still possible to evaluate the rise or fall in 'colony-like structures' counted over time. In single time point evaluation it is not thought feasible to subtract the many cell aggregates of less than colony size present in culture (Umbach & Spitzer, 1983).

Clumps would either remain constant or deteriorate. In the latter case, swelling of the dying cells may cause small clumps to 'grow' and make them detectable by automated counting. Consequently, cell death would cause disaggregation of the clumps. A decrease in the number of colonies is often seen after an early initial increase in the temporal growth pattern. Colonies formed in the meantime, conversely, increase the number counted after the fall, allowing detection of growth.

Still, additional criteria such as total area of colonies as proposed by Thomson *et al.* (1984) may prove to be useful. In the study of primary human tumours, of often poor quality, this method may have the disadvantage that when deterioration occurs, swelling of the cells will cause the total area to increase. This will not be fully corrected by the disaggregation of the clumps, clusters or colonies. A combination of the use of both total area and number of 'objects' greater than a certain biologically acceptable lower limit (e.g. 60 μm) might prove to give optimal information on *in vitro* tumour cell growth.

The disadvantages in the use of TGPs are mainly of a logistical nature. The main problem concerns the availability of tumour material. Often small biopsies or body fluids containing only a few tumour cells are offered for culture. This may give rise to problems in plating a growth test with as many as 24 dishes used by us, and even more frequently to problems for chemosensitivity testing with at least 18 dishes for each drug and control. Despite this requirement, more cultures prove to be evaluable for growth when using temporal growth patterns than in single time point assessment. While it is necessary to reduce the risk of infection, counting new dishes each time allows a fair interpretation of the growth pattern since the criterion of consistent increase over 3 countings is used. Thus it is very unlikely that dish to dish variability will be interpreted as consistent growth.

The growth positive rate of 25% as assessed by TGPs in this study seems to be lower than reported generally for ovarian carcinoma cultures (Williams *et al.*, 1983). This is explained by the rigid criteria for colony growth used in this study as opposed to a low threshold for colony formation of e.g. 5 colonies used in large studies (Sandbach *et al.*, 1982; Cowan & Von Hoff, 1983) as well as as a low number of cells (20) for a cell aggregate to be called a 'colony' by others (Kern *et al.*, 1982; Bertelsen *et al.*, 1984; Alonso, 1984). It should be noted especially that in the concept of TGPs it is not enough to see a sufficient number of colonies on *any* day, but that temporal increase should be consistent and reach 100%.

The fact that the mean time to peak is 20 days shows that Day 21 counts seems to be well timed.

However, the great range in time to peak shows that the right time for evaluation cannot be predicted for individual cultures for single time point assessment.

In conclusion, the use of TGPs may not make soft agar cultures available for routine use as a chemosensitivity test but this type of growth assessment can point out sample to sample variability in growth patterns and therefore show the right time for evaluation, potentially with additional parameters to conventional evaluation.

The authors wish to acknowledge the excellent technical assistance of O.E. Gellings, A.M.M. Aalders, C.N. Verrijp and J. Vedder.

### References

ALBERTS, D.S. CHEN, H.S.G., SOEHNLEN, B. & 4 others. (1980). In-vitro clonogenic assay for predicting response of ovarian cancer to chemotherapy. *Lancet*, **ii**, 340.

ALONSO, K. (1984). Human tumor stem cell assay: a prospective clinical trial. *Cancer*, **54**, 2475.

BERTELSEN, C.A., SONDAK, V.K., MANN, B.D., KORN, E.L. & KERN, D.H. (1984). Chemosensitivity testing of human solid tumors. *Cancer*, **53**, 1240.

COWAN, J.D. & VON HOFF, D.D. (1983). The human tumor cloning assay: an *in vitro* assay for anti-tumor activity in solid tumors. In *Cancer Chemotherapy*, Muggia, F.M. (ed) p. 103. Nijhoff: Boston.

HAMBURGER, A.W. & SALMON, S.E. (1977). Primary bioassay of human tumor stem cells. *Science*, **197**, 461.

HAMBURGER, A.W. & SALMON, S.E. (1980). Development of a bioassay for human myeloma colony-forming cells. In *Cloning of Human Tumor Stem Cells*, Salmon, S.E. (ed) p. 23. Alan R. Liss: New York.

HERMAN, C.J., PELGRIM, O.E., KIRKELS, W.J., DEBRUYNE, F.M.J. & VOOYS, G.P. (1983*a*). 'Viable' tumor cells in post-therapy biopsies: an application of human tumor clonogenic cells culture. *Arch. Path. Lab. Med.*, **107**, 81.

HERMAN, C.J., PELGRIM, O.E., KIRKELS, W.J. & 4 others. (1983*b*). In-use evaluation of the Omnicon automated tumor colony counter. *Cytometry*, **3**, 4339.

KERN, D.H., CAMPBELL, M.A., COCHRAN, A.J., BURK, M.W. & MORTON, D.L. (1982). Cloning of human solid tumors in soft agar. *Int. J. Cancer*, **30**, 725.

KIRKELS, W.J., PELGRIM, O.E., HOOGENBOOM, A.M.M. & 4 others. (1983). Patterns of tumor colony development over time in soft-agar culture. *Int. J. Cancer*, **32**, 399.

OZOLS, R.F., WILLSON, J.K.W., WELTZ, M.D. & 3 others. (1980). Inhibition of human ovarian cancer colony formation by adriamycin and its major metabolites. *Cancer Res.*, **40**, 4109.

SALMON, S.E. (1980). Application of the human tumor stem cell assay to new drug evaluation and screening. In *Cloning of Human Tumor Stem Cells*, Salmon, S.E. (ed) p. 291. Alan R. Liss: New York.

SANDBACH, J., VON HOFF, D.P., CLARK, G., CRUZ, A.B. & O'BRIEN, M. (1982). Direct cloning of human breast cancer in soft agar culture. *Cancer*, **50**, 1315.

THOMSON, S.P., MOON, T.E. & MEYSKENS, F.L. (1984). Kinetics of clonogenic melanoma cell proliferation and the limits on growth within a bilayer agar system. *J. Cell. Physiol.*, **121**, 114.

UMBACH, G. & SPITZER, G. (1983). 'Clumpogenic' v Clonogenic Assay (letter to the editor). *Lancet*, **ii**, 628.

VERHEIJEN, R.H.M., HERMAN, C.J. & KENEMANS, P. (1984). Applications of a human tumor clonogenic cell culture system in gynecologic oncology: review and personal experience. *Eur. J. Obstet. Gynecol. Repr. Biol.*, **17**, 43.

VON HOFF, D.D., HARRIS, G.J., JOHNSON, G. & GLAUBIGER, D. (1980). Initial experience with the human tumor stem cell assay system: potential and problems. In *Cloning of Human Tumor Stem Cells*, Salmon, S.E. (ed) p. 113. Alan R. Liss: New York.

VON HOFF, D.D., CASPER, J., BRADLEY, E. & 3 others. (1981). Association between tumor colony forming assay results and response of an individual patient's tumor to chemotherapy. *Am. J. Med.*, **70**, 1027.

VON HOFF, D.D., CLARK, G.M., STOGDILL, B.J. & 7 others. (1983). Prospective clinical trial of a human tumor cloning system. *Cancer Res.*, **43**, 1926.

WILLIAMS, T.J., LIEBER, M.M., PODRATZ, K.C. & MALKASIAN, G.D. (1983). Soft agar colony formation assay for in vitro testing of sensitivity to chemotherapy of gynecologic malignancies. *Am. J. Obstet. Gynecol.*, **145**, 940.